# Is the location of the needling point in sham acupuncture for tension-type headache associated with outcomes? A systematic review and network meta-analysis

**DOI:** 10.3389/fpain.2026.1874475

**Published:** 2026-08-06

**Authors:** Qiu-Yue Tu, Zi-Yi He, Jing-Sheng Huang, Ying-Shan Deng, Xin-Yu PENG, Yu-Feng Xie

**Affiliations:** 1 Department of Acupuncture and Moxibustion, Shenzhen Hospital (Futian) of Guangzhou University of Chinese Medicine, Shenzhen, China; 2 Department of Rehabilitation, the Zhongshan People’s Hospital, Guangdong, China; 3 Guangzhou University of Chinese Medicine, Guangzhou, China; 4 Shenzhen Pingshan Traditional Chinese Medicine Hospital, Shenzhen, China

**Keywords:** acupuncture, needling location, network meta-analysis, placebo control, sham acupuncture, tension-type headache

## Abstract

**Objective:**

In trials of acupuncture for tension-type headache (TTH), sham acupuncture may not be physiologically inert, and its design may influence the estimated specific effect of acupuncture. Sham acupuncture was classified into the following 2 types: (1) sham acupuncture needling at the same acupuncture points as those in the acupuncture group, referred to as sham acupuncture therapy (verum) (SATV) and (2) sham acupuncture needling at points different from those in the acupuncture group, referred to as sham acupuncture therapy (sham) (SATS). Therefore, we aimed to evaluate whether the needling point location in sham acupuncture is associated with treatment outcomes for TTH.

**Methods:**

Eight electronic databases were searched from inception to September 4, 2025. Randomized controlled trials (RCTs) comparing manual acupuncture with sham acupuncture or waiting-list control in adults with TTH diagnosed within the contemporaneous IHS/ICHD framework were eligible. Sham acupuncture was classified by needling location as SATV or SATS. The primary outcome was pain intensity; secondary outcomes were headache days and responder rate, defined as at least a 50% reduction in headache days. Random-effects frequentist network meta-analysis was performed after evaluating clinical similarity, transitivity, and statistical consistency. Certainty of evidence was assessed using the GRADE framework for network meta-analysis.

**Results:**

Six RCTs involving 1,055 participants were included. Compared with waiting-list control, acupuncture, SATV, and SATS were each associated with improvements in pain intensity, headache days, and responder rate. No statistically significant difference was detected between SATV and SATS for any outcome. Acupuncture did not differ significantly from either sham approach for pain intensity or headache days; however, acupuncture was associated with a higher responder rate than both SATV and SATS. The certainty of network evidence ranged from moderate to low for continuous outcomes and from high to low for responder rate, mainly because of risk of bias and imprecision.

**Conclusion:**

Within the available sparse network, the anatomical location of sham acupuncture alone did not explain differences in TTH outcomes. Sham acupuncture procedures used in previous TTH trials may carry physiological and contextual activity, regardless of whether they are delivered at the same acupoints or at different points. Future trials should move beyond simply debating “where to needle” and should quantify and report the full sham-acupuncture dose, including insertion depth, stimulation intensity, retention time, frequency, patient information, and blinding assessment.

**Systematic Review Registration:**

https://www.crd.york.ac.uk/PROSPERO/view/CRD420251135928, identifier CRD420251135928.

## Introduction

Tension-type headache (TTH) is one of the most prevalent neurological disorders worldwide and contributes substantially to population-level disability and health-care use (1–3). It is typically characterized by mild to moderate bilateral tightening, pressing, or dull pain, often localized in the temporal regions, with possible radiation to the forehead and occipital area (4). Because TTH is often recurrent or chronic, safe and acceptable non-pharmacological approaches remain clinically important.

Acupuncture is widely used as a non-pharmacological intervention for TTH (5). Previous systematic reviews have suggested that acupuncture may reduce headache frequency or improve symptoms, particularly when compared with no treatment or usual care. However, the estimated advantage of verum acupuncture over sham acupuncture has been smaller and less consistent. This pattern has raised a central methodological question in acupuncture trials: whether the sham procedure is an inert placebo or an active intervention with physiological and contextual effects (6–8).

Sham acupuncture is not a single, uniform control. Trials have used non-penetrating devices, blunt needles, superficial insertion, minimal stimulation, or needling at non-acupuncture points. A particularly important design feature is the location of the sham procedure (9, 10). Some studies apply sham acupuncture at the same acupoints as verum acupuncture but with reduced or non-penetrating stimulation, whereas others apply sham procedures at points different from those used for verum acupuncture. These two designs may differ in biological plausibility, participant credibility, and risk of inadvertently producing therapeutic effects.

Conventional pairwise meta-analyses usually pool different sham acupuncture procedures as one comparator, which may obscure clinically meaningful differences in control design. Therefore, we conducted a systematic review and network meta-analysis to compare acupuncture, sham acupuncture needling at the same acupuncture points as those in the acupuncture group, referred to as sham acupuncture therapy (verum) (SATV), sham acupuncture needling at points different from those in the acupuncture group, referred to as sham acupuncture therapy (sham) (SATS), and waiting-list control in adults with TTH. Our aim was not only to estimate clinical effects but also to clarify whether the needling location of sham acupuncture is associated with trial outcomes.

## Methods

The protocol was registered with PROSPERO (registration number: ID CRD420251135928). This NMA was reported in accordance with the relevant extension of the Preferred Reporting Items for Systematic Reviews and Meta-analyses (PRISMA) reporting guideline.

### Eligibility criteria

**Figure 1:**
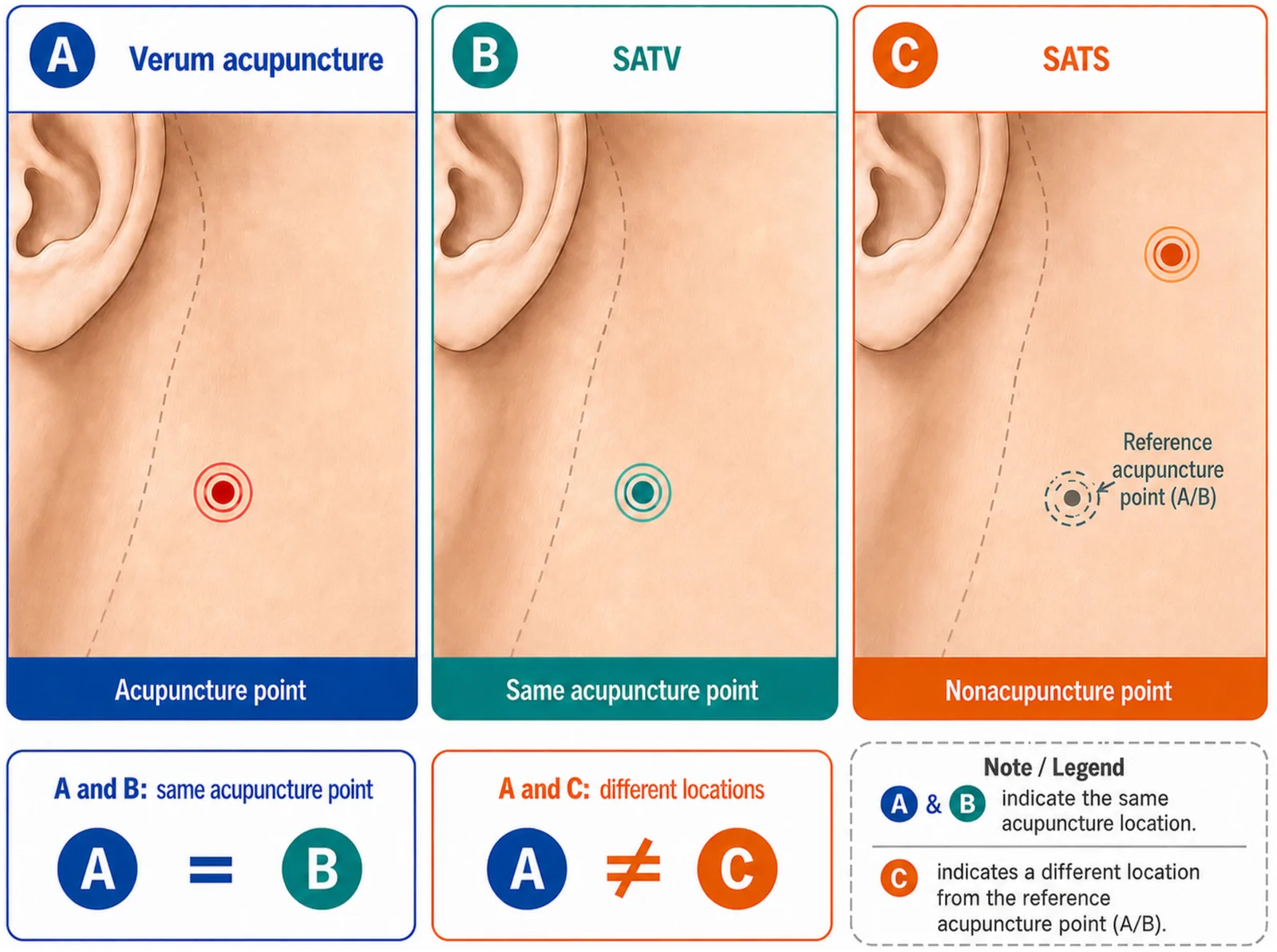
Schematic illustration of sham acupuncture conditions. SATV, sham acupuncture needling at the same acupuncture points as those in the acupuncture group, referred to as sham acupuncture therapy (verum); SATS, sham acupuncture needling at points different from those in the acupuncture group, referred to as sham acupuncture therapy (sham).

1. Participants: Adult patients diagnosed with TTH were included in this study. There were no restrictions regarding sex, age, or nationality. However, individuals with other primary or secondary headache disorders were excluded. To improve diagnostic comparability, we preferentially included RCTs in which TTH was diagnosed according to the contemporaneous International Headache Society/International Classification of Headache Disorders (IHS/ICHD) framework. Because the included trials were conducted across different periods, the exact diagnostic version was not identical across all studies; however, all eligible studies were based on the IHS/ICHD diagnostic framework for TTH.
2. Interventions and Comparators: The interventions of interest were acupuncture, sham acupuncture, and a waiting list control. Acupuncture was defined as manual acupuncture involving needle insertion. Sham acupuncture procedures differed in technique (e.g., superficial insertion, placebo devices) and were classified by needling location: ① SATV: sham acupuncture needling at the same acupuncture points as those in the acupuncture group, referred to as sham acupuncture therapy (verum); ② SATS: sham acupuncture needling at points different from those in the acupuncture group, referred to as sham acupuncture therapy (sham) (Figure 1). The waiting list group was included as an intervention and comparator to form a connected loop on the network plot and to compare the real-world effectiveness of the acupuncture and sham acupuncture groups.
3. Outcomes: The primary outcome was pain intensity, as measured by the visual analog scale (VAS), numeric rating scale (*N*RS), or other validated outcome measure. Secondary outcomes included the number of headache days per defined period and the responder rate, with response defined as achieving at least a 50% reduction in headache days from baseline.
4. Study design: All Prospective RCTs comparing manual acupuncture with sham acupuncture, waiting list, or both were included.

### Information sources and search strategy

September 4, 2025 represented the last date on which the prespecified full search, screening, eligibility assessment, data extraction, and synthesis workflow was finalized. During revision, we additionally checked for subsequently published studies and did not identify any newly published completed RCTs that clearly met all prespecified eligibility criteria; therefore, the evidence network and conclusions remained unchanged.

Eight electronic databases were searched on September 4, 2025: China National Knowledge Infrastructure (CNKI), Wanfang Data, Chongqing VIP, Chinese Biomedical Literature Database (CBM), MEDLINE, Embase, Cochrane Central Register of Controlled Trials (CENTRAL), and the Allied and Complementary Medicine Database (AMED). To identify further relevant studies, the reference lists of all included trials and relevant review articles were manually screened. The search strategy for each database is provided in Supplementary S1.

### Study selection and data extraction

Two researchers independently performed the study screening and data extraction. Discrepancies were resolved through discussion or, when necessary, by consulting a third reviewer. Studies were initially screened based on titles and abstracts to determine eligibility for inclusion. Full-text articles were subsequently retrieved and reviewed for potentially eligible studies. From each included study, the following data were extracted: first author, publication year, country, patient characteristics, sample size, details of acupuncture and sham acupuncture interventions, treatment duration, and primary/secondary outcome measures. If the relevant information was ambiguous, the corresponding author was contacted via email to request additional details.

### Risk of bias assessment

The quality assessment of included studies was independently performed by two researchers using the Cochrane Risk of Bias 2 tool (11). Researchers responded to the tool's signaling questions according to its algorithm, which then automatically generated a risk-of-bias judgment (“low risk,” “some concerns,” or “high risk”) for each domain. The results were synthesized into a risk-of-bias summary figure. Any discrepancies between reviewers were resolved through discussion or by consulting a third researcher.

### Data analysis and synthesis

The main characteristics of the included studies were first summarized descriptively. For direct evidence, pairwise meta-analyses were conducted using Review Manager 5.4 (Cochrane, London, UK) to examine consistency in statistical significance with the network meta-analysis (NMA) results. For indirect or mixed evidence, a frequentist NMA was performed using the network package in Stata/MP 16.1 (StataCorp LLC, College Station, TX, USA), following an evaluation of similarity, consistency, and transitivity assumptions. Consistency between direct and indirect evidence was specifically assessed using a global approach (design-by-treatment interaction model) and local approaches (node-splitting method and loop inconsistency test), with *p* < 0.05 indicating statistically significant inconsistency.

A four-node network diagram was constructed for each outcome, comprising four intervention nodes: Acupuncture therapy (AT), SATV, SATS, and waiting list. Node sizes were proportional to the sample size of each intervention group, and the thickness of the connecting lines reflected the number of trials available for direct comparisons. A league table was generated to display all pairwise comparison results, and the Surface Under the Cumulative Ranking Curve (SUCRA) was calculated to provide a probabilistic ranking of intervention efficacy.

Due to expected clinical heterogeneity across studies, random-effects models were applied for both pairwise meta-analysis and NMA. Continuous outcomes (pain intensity and headache days) were analyzed using standardized mean difference (SMD) with 95% confidence intervals (CI), given variability in measurement instruments. For the dichotomous outcome (response rate), risk ratio (RR) with 95% CI was used. Results were interpreted as statistically non-significant if the 95% CI for SMD included 0, or for RR included 1. If a sufficient number of studies (*n* ≥ 10) were included in the analysis, funnel plot symmetry and Egger's test were used to assess potential publication bias.

### Certainty of evidence assessment

The certainty of evidence for the effect estimates was assessed using the Grading of Recommendations, Assessment, Development, and Evaluations (GRADE) approach (12, 13). First, the quality of direct evidence was evaluated based on risk of bias, inconsistency, indirectness, and publication bias. Subsequently, the certainty of indirect estimates was determined by taking the lower rating of the two contributing direct comparisons within the dominant first-order loop, while also considering intransitivity. Finally, for the NMA estimate, the certainty level was established by selecting the higher rating from the corresponding direct and indirect evidence, then further evaluating incoherence and imprecision. For each outcome comparison, the overall certainty of evidence was categorized as high, moderate, low, or very low.

## Results

### Study selection

**Figure 2:**
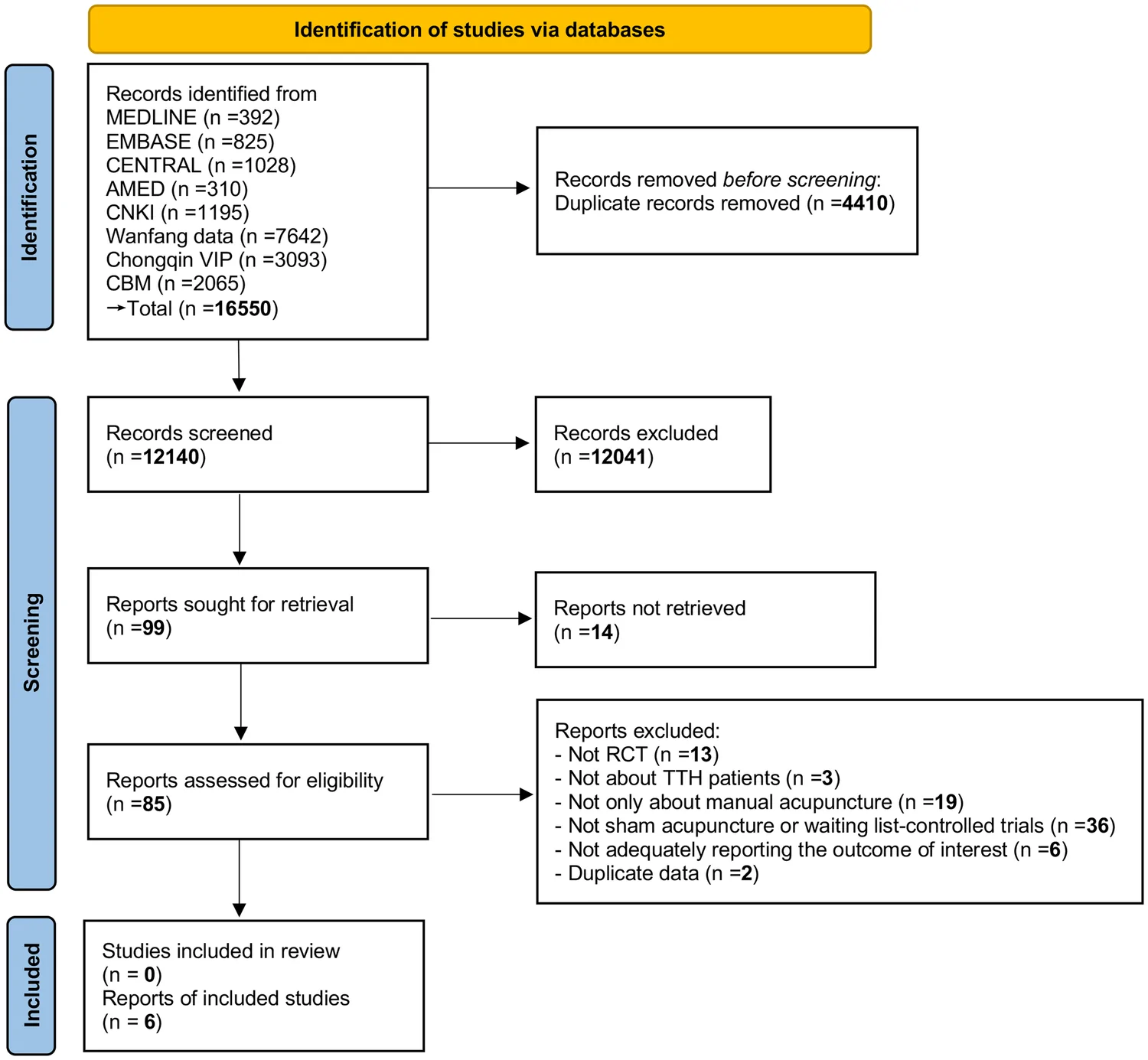
Flow diagram of the literature screening and selection processes. CENTRAL, the cochrane central register of controlled trials; AMED indicates allied and complementary medicine database; RCT, randomized clinical trial. TTH, Tension-Type Headache.

A total of 16,550 studies were identified through database searches (Supplementary S1). After deduplication, title and abstract screening, and full-text retrieval, 85 studies were assessed in full text. As a result, 79 studies were excluded: non-RCT design (*n* = 13), not about TTH patients (*n* = 3), not only about manual acupuncture (*n* = 19), not sham acupuncture or waiting list-controlled trials (*n* = 36), not adequately reporting the outcome of interest (*n* = 6), and duplicate data (*n* = 2) (Supplementary S2). Finally, 6 studies involving 1,055 subjects were included (14–19) (Figure 2).

### Study characteristics

A total of 4 studies were conducted in Germany (15, 16, 18, 19), and 1 study was conducted each in China (17), the United Kingdom (14). A total of 5 studies were two-arm RCTs comparing acupuncture with sham acupuncture (14, 16–19). 1 study was a three-arm RCT comparing acupuncture, sham acupuncture, and waiting list groups (15). For the needling points for sham acupuncture, 3 studies performed SATV (17–19), and 3 studies performed SATS (14–16). For the technique of sham acupuncture, 4 studies used superficial needling (15–17, 19), 1 study used a blunt needle to touch the skin (18), and 1 study used a blunted cocktail-stick (14). Pain intensity was evaluated in 5 studies (14–19), headache days were evaluated in 5 studies (14–17, 19), and response rate was evaluated in 4 studies (14–17) (Table 1 and Supplementary S3).

**Table 1:** Characteristics of included studies.

Source (country)	Mean (SD) age, y	AT (sample size)	Sham AT (sample size)	Waiting list (sample size)	Treatment duration	Outcomes of interest	Time point included in the analysis
AR White, 2000 (UK)	AT: 49.8 (2.9)/Sham AT: 48.2 (2.9)	25	SATS:a blunted cocktail-stick at nonacupuncture points (25)	NA	5 weeks	Pain intensity: VAS/Headache days/ Response rate	5 weeks
Dieter Melchart, 2005 (Germany)	AT: 42.3 (13.5)/Sham AT: 43.4 (12.9)/WL: 42.8 (13.2)	132	SATS: superficial needling at nonacupuncture points (63）	75	8 weeks	Pain intensity: VAS/Headache days/ Response rate	8 weeks
Heinz G. Endres, 2007 (Germany)	AT:39.2 (11.4)/Sham AT:38.9 (12.2)	209	SATS: superficial needling at nonacupuncture points (200）	NA	6 weeks	Headache days/ Response rate	6 weeks
Hui Zheng, 2022 (China)	AT:43.0 (12.5)/Sham AT:43.2 (12.8)	110	SATV: superficial needling at same acupuncture points used in the AT group (108)	NA	8 weeks	Pain intensity: VAS/Headache days/ Response rate	8 weeks
Matthias Karst, 2000 (Germany)	AT:50.4 (13.5)/Sham AT: 47.3 (16.5)	21	SATV: a blunt needle touches the skin at same acupuncture points used in the AT group (18)	NA	5 weeks	Pain intensity: VAS	5 weeks
M Karst, 2001 (Germany)	AT:47.9 (13.8)/Sham AT: 48.2 (14.6)	34	SATV: superficial needling at same acupuncture points used in the AT group (35)	NA	5 weeks	Pain intensity: VAS/Headache days	5 weeks

AT, acupuncture therapy; SATV, sham acupuncture needling at the same acupuncture points as those in the acupuncture group, referred to as sham acupuncture therapy (verum); SATS, sham acupuncture needling at points different from those in the acupuncture group, referred to as sham acupuncture therapy (sham); NA, not applicable; VAS, visual analog scale.

**Figure 3:**
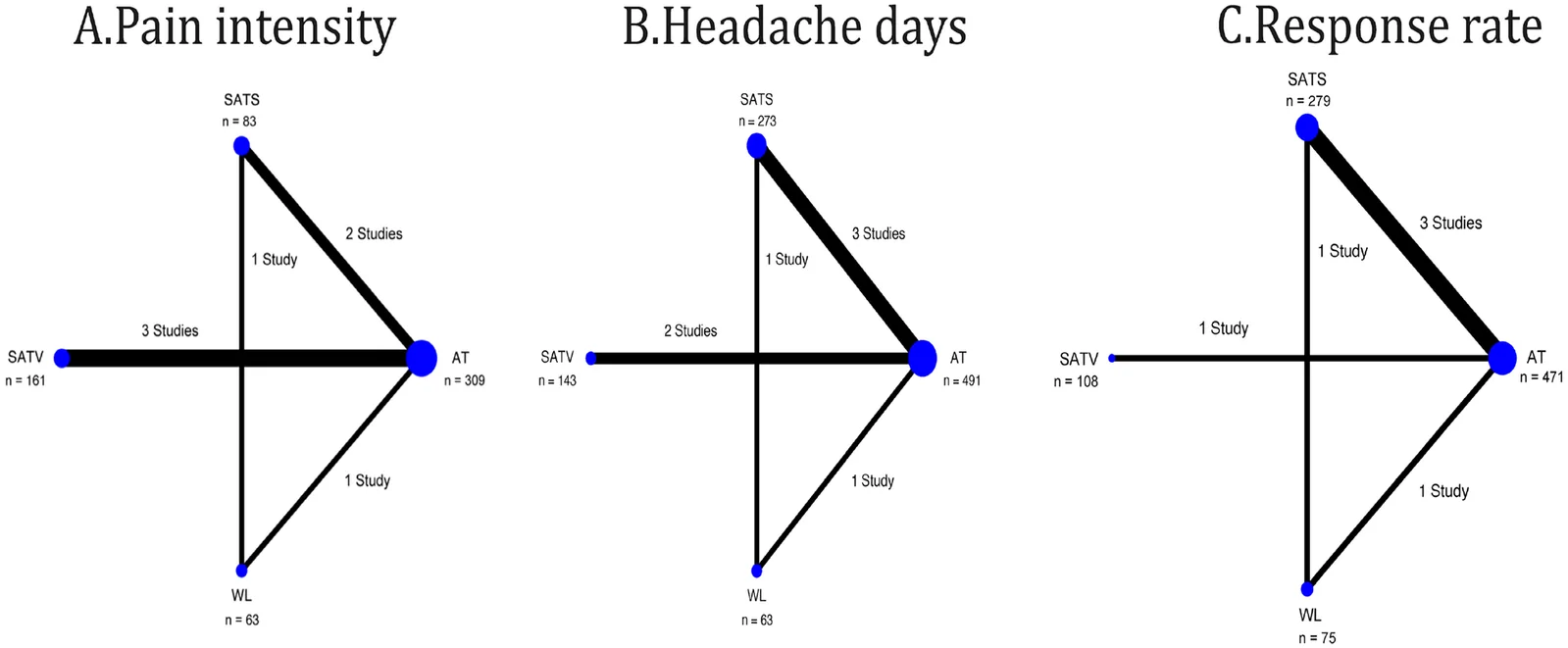
Network maps for pain intensity, headache days, and response rate. AT, acupuncture therapy; SATV, sham acupuncture needling at the same acupuncture points as those in the acupuncture group, referred to as sham acupuncture therapy (verum); SATS, sham acupuncture needling at points different from those in the acupuncture group, referred to as sham acupuncture therapy (sham); WL, waiting list.

There were no statistical inconsistencies according to the global (Pain intensity, *χ*^2^ = 1.32; *P* = 0.2513; Headache days, *χ*^2^ = 0.16; *P* = 0.6921; Response rate, *χ*^2^ = 0.16; *P* = 0.6934) and local approaches (Supplementary S4). Pain intensity, headache days, and response rate each had one closed loop, and testing revealed that the *p*-values were all greater than 0.05, indicating good consistency within the closed loops (Supplementary S5). Figure 3 shows the network geometry of pain intensity, headache days, and response rate.

### Risk of bias within studies

**Figure 4:**
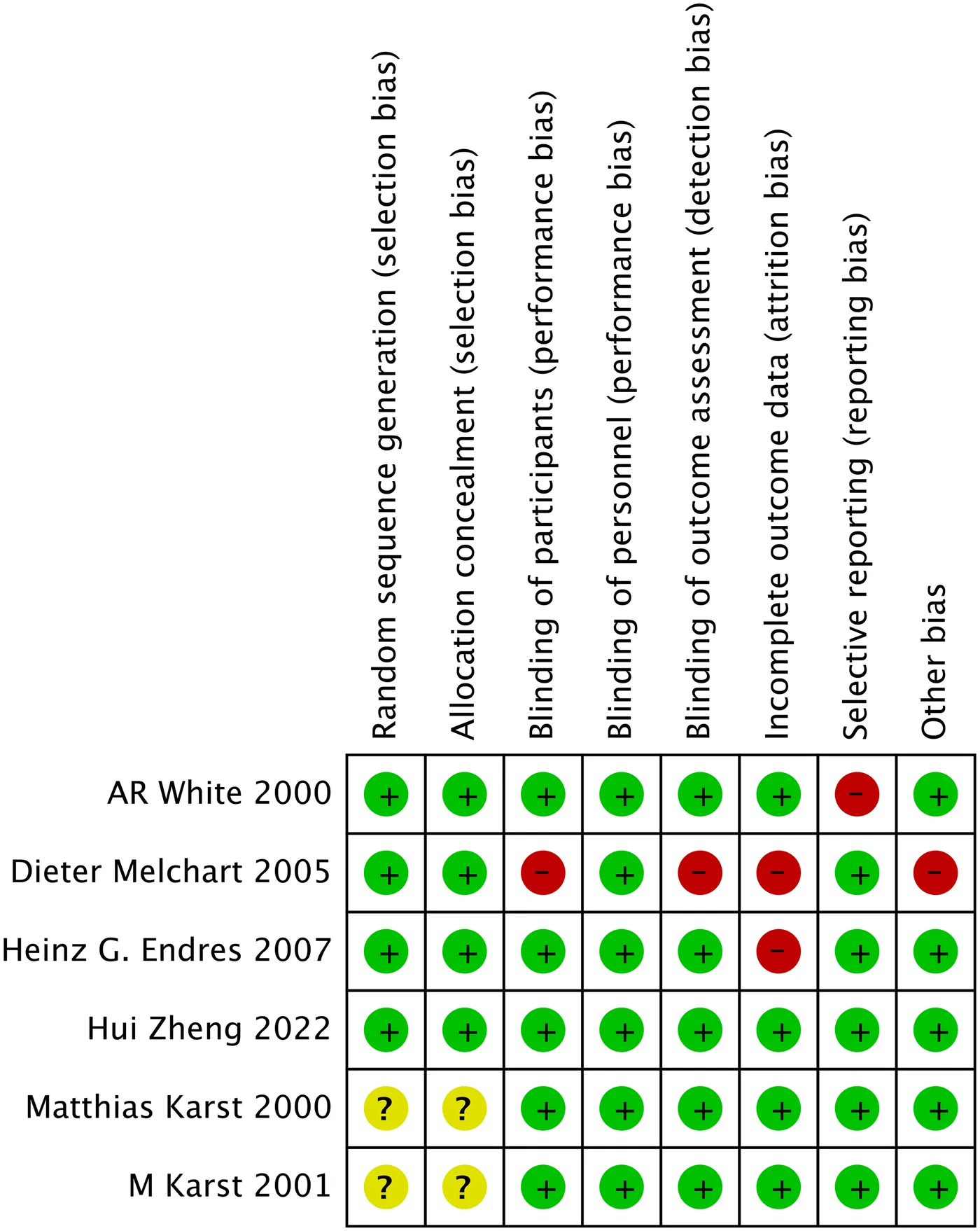
Risk of bias summary for all included studies. Low risk of bias, some concerns, and high risk of bias, respectively, are represented with the following symbols: “+”, “?”, and “−”.

A total of 4 studies (14–17) appropriately generated random sequences using statistical software methods, and 2 studies (18, 19) were evaluated as having an unclear risk of bias due to insufficient reporting information on random sequence generation and allocation concealment. In 1 study (15), blinding of participants was not possible due to waiting list controls, and outcome data were collected via patient-reported questionnaires; thus, we rated them as having a high risk of performance and detection bias. Blinding of acupuncturists was not possible in any of the trials; however, because the acupuncturists were not involved in outcome assessment, this was judged not to affect the results. 2 studies (15, 16) did not perform intent-to-treat analysis and were thus evaluated as having a high risk of attrition bias. 1 study (14) did not report the quantity of analgesic consumption and was assessed as having a high risk of reporting bias. 1 study (15) was rated as high risk of other bias due to significant differences in baseline clinical characteristics between groups (Figure 4).

### Data analysis: pain intensity

**Figure 5:**
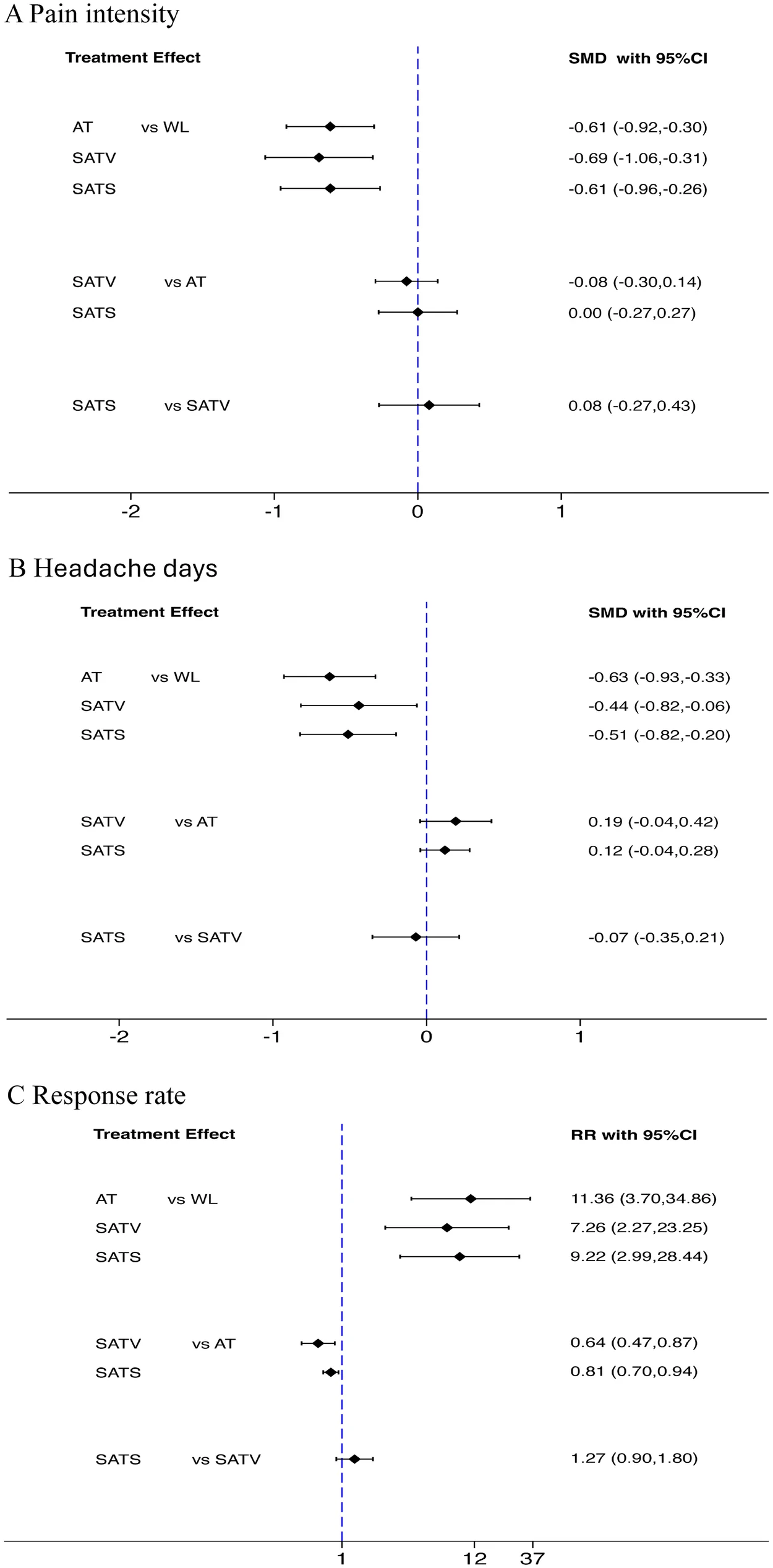
Interval plots for **(A)** pain intensity, **(B)** headache days and **(C)** response rate. AT, acupuncture therapy; SATV, sham acupuncture needling at the same acupuncture points as those in the acupuncture group, referred to as sham acupuncture therapy (verum); SATS, sham acupuncture needling at points different from those in the acupuncture group, referred to as sham acupuncture therapy (sham); WL, waiting list; SMD, standardized mean difference; RR, risk ratio.

According to NMA results, compared with the waiting list, acupuncture (SMD −0.61, 95% CI −0.92 to −0.30), SATV (SMD −0.69, 95% CI −1.06 to −0.31), and SATS (SMD −0.61, 95% CI −0.96 to −0.26) all significantly reduced pain intensity. No statistically significant difference was detected between SATV and acupuncture (SMD −0.08, 95% CI −0.30 to 0.14), SATS and acupuncture (SMD 0.00, 95% CI −0.27 to 0.27), or SATS and SATV (SMD 0.08, 95% CI −0.27 to 0.43) (Figure 5A). The findings from the pairwise meta-analysis were consistent with the NMA (Table 2). The SUCRA plot showed that SATV was ranked first (58.9%), followed by SATS (28.9%), acupuncture (12.2%), and waiting list (0%) (Supplementary S6). Because the pain-intensity network was sparse and the relative effect estimates between acupuncture and sham approaches were not statistically significant, the SUCRA ranking should be interpreted cautiously as descriptive rather than definitive and should not be over-read as evidence that SATV is clinically superior to verum acupuncture. Publication bias could not be assessed using funnel plots or Egger's test due to the limited number of studies (*n* = 5) included in the NMA.

**Table 2:** League table for pairwise meta-analysis (right upper part) and network meta-analysis (left lower part) estimates.

Outcome	Comparison	Pairwise meta-analysis, effect estimate (95% CI)	Network meta-analysis, effect estimate (95% CI)
**Pain intensity, SMD**	AT vs. WL	**−0.58** (**−0.89, −0.27)**	**−0.61** (**−0.92, −0.30)**
	SATV vs. WL	—	**−0.69** (**−1.06, −0.31)**
	SATS vs. WL	**−0.06** (**−1.02, −0.29)**	**−0.61** (**−0.96, −0.26)**
	SATV vs. AT	−0.08 (−0.30, 0.14)	−0.08 (−0.30, 0.14)
	SATS vs. AT	−0.02 (−0.36, 0.31)	0.00 (−0.27, 0.27)
	SATS vs. SATV	—	0.08 (−0.27, 0.43)
**Headache days, SMD**	AT vs. WL	**−0.61** (**−0.92, −0.30)**	**−0.63** (**−0.93, −0.33)**
	SATV vs. WL	—	**−0.44** (**−0.82, −0.06)**
	SATS vs. WL	**−0.56** (**−0.93, −0.20)**	**−0.51** (**−0.82, −0.20)**
	SATV vs. AT	0.19 (−0.04, 0.42)	0.19 (−0.04, 0.42)
	SATS vs. AT	0.12 (−0.04, 0.28)	0.12 (−0.04, 0.28)
	SATS vs. SATV	—	−0.07 (−0.35, 0.21)
**Response rate, RR**	AT vs. WL	**11.55** (**3.75, 35.55)**	**11.36** (**3.70, 34.86)**
	SATV vs. WL	—	**7.26** (**2.27, 23.25)**
	SATS vs. WL	**8.73** (**2.74, 27.82)**	**9.22** (**2.99, 28.44)**
	SATV vs. AT	**0.64** (**0.41, 0.88)**	**0.64** (**0.47, 0.87)**
	SATS vs. AT	**0.81** (**0.70, 0.93)**	**0.81** (**0.70, 0.94)**
	SATS vs. SATV	—	1.27 (0.90, 1.80)

AT, acupuncture therapy; SATV, sham acupuncture needling at the same acupuncture points as those in the acupuncture group, referred to as sham acupuncture therapy (verum); SATS, sham acupuncture needling at points different from those in the acupuncture group, referred to as sham acupuncture therapy (sham); WL, waiting list; SMD, standardized mean difference; RR, risk ratio; CI, confidence interval. All estimates are expressed as the first-listed treatment relative to the second-listed treatment. For pain intensity and headache days, negative SMD values favor the first-listed treatment. For response rate, RR values greater than 1 favor the first-listed treatment.

Bold values indicate statistically significant differences, defined as 95% CIs excluding 0 for SMDs and excluding 1 for RRs.

An em dash indicates that no direct pairwise evidence was available.

### Data analysis: headache days

According to NMA results, compared with the waiting list, acupuncture (SMD −0.63, 95% CI −0.93 to −0.33), SATV (SMD −0.44, 95% CI −0.82 to −0.06), and SATS (SMD −0.51, 95% CI −0.82 to −0.20) all significantly reduced headache days. No statistically significant difference was detected between SATV and acupuncture (SMD 0.19, 95% CI −0.04 to 0.42), SATS and acupuncture (SMD 0.12, 95% CI −0.04 to 0.28), or SATS and SATV (SMD −0.07, 95% CI −0.35 to 0.21) (Figure 5B). The findings from the pairwise meta-analysis were consistent with the NMA (Table 2). The SUCRA plot showed that acupuncture was ranked first (87.6%), followed by SATS (6.4%), SATV (6%), and waiting list (0%) (Supplementary S6). Publication bias could not be assessed using funnel plots or Egger's test due to the limited number of studies (*n* = 5) included in the NMA.

### Data analysis: response rate

According to NMA results, compared with the waiting list, acupuncture (RR 11.36, 95% CI 3.70–34.86), SATV (RR 7.26, 95% CI 2.27–23.25), and SATS (RR 9.22, 95% CI 2.99–28.44) were all associated with significantly higher responder rates. Compared with acupuncture, both SATV (RR 0.64, 95% CI 0.47–0.87) and SATS (RR 0.81, 95% CI 0.70–0.94) showed lower responder rates. No statistically significant difference was detected between SATV and SATS (RR 1.27, 95% CI 0.90–1.80) (Figure 5C). The findings from the pairwise meta-analysis were consistent with the NMA (Table 2). The SUCRA plot showed that acupuncture was ranked first (99.5%), followed by SATS (0.3%), SATV (0.2%), and waiting list (0%) (Supplementary S6). Publication bias could not be assessed using funnel plots or Egger's test due to the limited number of studies (*n* = 4) included in the NMA.

### Certainty of evidence

For pain intensity and headache days, the certainty of direct and indirect evidence was rated as moderate, downgraded due to the risk of bias in individual studies. The certainty of NMA evidence was moderate or low, and the reason for further downgrading was imprecision in the effect estimates. For the response rate, the certainty of direct and indirect evidence was high or moderate, downgraded due to the risk of bias in individual studies. The certainty of NMA evidence was high, moderate, or low, downgraded due to the risk of bias and imprecision in the effect estimates (Supplementary S7).

## Discussion

This systematic review and network meta-analysis examined whether the needling location used in sham acupuncture was associated with clinical outcomes in randomized trials of acupuncture for TTH. The main finding was that no statistically significant difference was detected between SATV and SATS for pain intensity, headache days, or responder rate. Compared with waiting-list control, verum acupuncture, SATV, and SATS were all associated with improvements in pain intensity, headache days, and responder rate. Verum acupuncture did not differ significantly from either SATV or SATS for pain intensity and headache days; however, it was associated with a higher responder rate than both sham acupuncture approaches. These findings suggest that the location of sham needling alone may not be the main factor determining clinical outcomes in TTH acupuncture trials.

A key issue raised by these findings is whether the observed effects should be interpreted as acupuncture-specific effects or as effects produced by any credible needling-related intervention. Our results do not support an interpretation that all observed benefits are attributable solely to the specific effects of verum acupuncture. Because verum acupuncture, SATV, and SATS were all superior to waiting-list control, clinical improvements in TTH trials may be partly driven by components shared across credible acupuncture-related interventions. These shared components may include skin contact or needle insertion, somatosensory stimulation, repeated clinical attention, patient-practitioner interaction, treatment expectation, and the therapeutic ritual of acupuncture (10, 20–22). This interpretation is particularly relevant for patient-reported outcomes such as pain intensity and headache days, which may be sensitive to contextual and non-specific treatment effects.

However, the present findings should not be interpreted as evidence that verum acupuncture has no specific effect. Although verum acupuncture was not significantly different from SATV or SATS for pain intensity and headache days, it showed a significant advantage in responder rate, defined as at least a 50% reduction in headache days. This outcome represents a clinically meaningful threshold rather than a small continuous change in symptoms. Therefore, the higher responder rate observed with verum acupuncture suggests that specific components of the complete verum acupuncture intervention may contribute to achieving substantial clinical improvement. These components may include acupuncture point selection, needling depth, manual stimulation, *de qi* sensation, needle retention time, treatment frequency, and the cumulative dose of treatment (23, 24). In other words, verum acupuncture may not be clearly distinguishable from sham acupuncture for modest changes in continuous symptom scores, but it may have greater potential to help a proportion of patients reach a clinically meaningful level of improvement.

At the trial level, sham procedures provided only partial control of nonspecific effects. Formal assessment of treatment expectancy, credibility of the sham procedure, practitioner attitude, or blinding success was limited or inconsistently reported across the included RCTs. Therefore, residual contextual and nonspecific effects could not be fully excluded.

The present results are consistent with previous evidence showing that the estimated efficacy of acupuncture depends strongly on the type of control group. Large individual patient data meta-analyses of chronic pain have shown that acupuncture is superior to both sham acupuncture and no-acupuncture controls, but the difference between acupuncture and sham acupuncture is generally smaller than the difference between acupuncture and no-treatment or waiting-list controls (10, 20)^.^ This pattern suggests that acupuncture effects are likely to include both specific effects of the verum intervention and non-specific or contextual effects shared with sham procedures. In TTH specifically, the Cochrane review suggested that acupuncture may be effective for frequent episodic or chronic TTH, but the distinction between acupuncture-specific effects and effects related to sham or contextual interventions remains difficult to determine (25).

The absence of a significant difference between SATV and SATS also indicates that whether sham needling is performed at the same acupuncture points as verum acupuncture or at different points may be less important than the overall physiological and contextual activity of the sham procedure. SATV may not be inert because stimulation at the same acupuncture points, even if superficial or non-penetrating, may still provide somatosensory input and may partially reproduce elements of the verum acupuncture ritual. SATS may also not be inert because superficial needling, skin touch, or stimulation at non-indicated points can still activate cutaneous afferents, induce local physiological responses, and generate credible treatment expectations (21, 22, 26). Therefore, both SATV and SATS should be regarded as active comparators with potential physiological and contextual effects rather than as pure placebo controls.

These findings also suggest that future acupuncture trials should move beyond simply asking whether sham needling is performed at the same or different points. Instead, sham acupuncture should be described and quantified as a complex intervention. Future trials should report the anatomical location of sham needling, whether the skin is penetrated, insertion depth, needle manipulation, sensory stimulation, elicitation or avoidance of de qi, retention time, treatment frequency, practitioner-patient interaction, information given to participants, credibility of the sham procedure, and success of blinding. Such reporting would allow future reviews to better distinguish acupuncture-specific effects from the broader effects of credible needling-related interventions. This recommendation is consistent with the ACURATE checklist, which emphasizes clear reporting of sham needling procedures to improve replicability and appraisal of sham-controlled acupuncture trials (26).

To the best of our knowledge, this is the first network meta-analysis focusing specifically on whether the location of sham needling is associated with outcomes in acupuncture trials for TTH. Its main contribution is not to prove that sham acupuncture is equivalent to verum acupuncture, but to show that the anatomical location of sham needling alone does not appear to explain outcome differences in the available evidence. The findings highlight the need to interpret sham acupuncture controls cautiously and to consider the full dose of sham procedures, including both physiological stimulation and contextual treatment components.

## Limitations

Several limitations should be acknowledged. First, only six RCTs were included, and the evidence networks were sparse. Therefore, the absence of statistically significant differences between SATV and SATS should not be interpreted as proof of equivalence. Second, sham acupuncture procedures differed across trials in penetration, stimulation method, retention time, number of sessions, and credibility, which may have influenced the estimated effects. Third, several outcomes were patient-reported and may be particularly sensitive to expectation, blinding, and patient-practitioner interaction. Fourth, the exact version of the TTH diagnostic criteria was not identical across all included study periods, which may have introduced some clinical heterogeneity. Fifth, most included trials were single-blinded because practitioner blinding is generally not feasible in acupuncture trials. Moreover, formal reporting of treatment expectancy, credibility assessment, practitioner-related contextual factors, and blinding success was limited; therefore, residual nonspecific effects could not be fully evaluated. Finally, SUCRA rankings should be interpreted cautiously because treatment rankings can be unstable in sparse networks and should not be regarded as definitive evidence of superiority.

## Conclusions

In conclusion, the current evidence suggests that the clinical effects observed in acupuncture trials for TTH cannot be attributed solely to the specific effects of verum acupuncture, nor can they be explained simply by whether sham acupuncture is performed at the same or different points. Verum acupuncture, SATV, and SATS were all associated with better outcomes than waiting-list control, indicating that credible needling-related interventions may influence TTH outcomes through both physiological and contextual mechanisms. Nevertheless, the higher responder rate associated with verum acupuncture suggests that specific components of the complete acupuncture intervention may contribute to clinically meaningful improvement. Future trials should standardize and transparently report sham acupuncture procedures to clarify the relative contributions of acupuncture-specific and non-specific effects.

## Data Availability

The original contributions presented in the study are included in the article/Supplementary Material, further inquiries can be directed to the corresponding author.

## References

[1] Wijeratne T Oh J Kim S Yim Y Kim MS Shin JI. Global, regional, and national burden of headache disorders, 1990–2021, with forecasts to 2050: a global burden of disease study 2021. Cell Rep Med. (2025) 6:102348. 10.1016/j.xcrm.2025.102348 40972580 PMC12629824

[2] Hus⊘y AK Xu YY Steinmetz JD Aalipour MA Aalruz H Abdulah DM. Global, regional, and national burden of headache disorders, 1990–2023: a systematic analysis for the global burden of disease study 2023. Lancet Neurol. (2025) 24(12):1005–15. 10.1016/S1474-4422(25)00402-8 41240916 PMC12612381

[3] Husøy AK Yu S Liu R Herekar AA Ahmed B Herekar AD. The global prevalence of headache disorders of public-health importance: a meta-analysis of population-based individual participant data from 41,614 adults from 17 countries. J Headache Pain. (2025) 26(1):204. 10.1186/s10194-025-02142-9 41057756 PMC12502191

[4] Vos T Barber RM Bell B Bertozzi-Villa A Biryukov S Bolliger I. Global, regional, and national incidence, prevalence, and years lived with disability for 301 acute and chronic diseases and injuries in 188 countries, 1990–2013: a systematic analysis for the global burden of disease study 2013. Lancet. (2015) 386(9995):743–800. 10.1016/S0140-6736(15)60692-4 26063472 PMC4561509

[5] Krøll LS Callesen HE Carlsen LN Birkefoss K Beier D Christensen HW. Manual joint mobilisation techniques, supervised physical activity, psychological treatment, acupuncture and patient education for patients with tension-type headache. A systematic review and meta-analysis. J Headache Pain. (2021) 22(1):96. 10.1186/s10194-021-01298-4 34418953 PMC8379845

[6] Davis MA Kononowech RW Rolin SA Spierings EL. Acupuncture for tension-type headache: a meta-analysis of randomized, controlled trials. J Pain. (2008) 9(8):667–77. 10.1016/j.jpain.2008.03.011 18499526

[7] Hao XA Xue CC Dong L Zheng Z. Factors associated with conflicting findings on acupuncture for tension-type headache: qualitative and quantitative analyses. J Altern Complement Med. (2013) 19(4):285–97. 10.1089/acm.2011.0914 23075410

[8] Birch S Hammerschlag R Trinh K Zaslawski C. The non-specific effects of acupuncture treatment: when and how to control for them clinical. Acupunct Orient Med. (2002) 3(1):20–5. 10.1054/caom.2001.0118

[9] Birch S, Alraek T, Kim KH, Lee MS. *Placebo-Controlled Trials in Acupuncture: Problems and Solutions.* Singapore: Springer Singapore (2016) p. 55–64. 10.1007/978-981-10-2290-6_4

[10] MacPherson H Vertosick E Lewith G Linde K Sherman KJ Witt CM. Influence of control group on effect size in trials of acupuncture for chronic pain: a secondary analysis of an individual patient data meta-analysis. PLoS One. (2014) 9(4):e93739. 10.1371/journal.pone.0093739 24705624 PMC3976298

[11] Sterne JAC Savović J Page MJ Elbers RG Blencowe NS Boutron I. Rob 2: a revised tool for assessing risk of bias in randomised trials. Br Med J. (2019) 366:l4898. 10.1136/bmj.l4898 31462531

[12] Puhan MA Schünemann HJ Murad MH Li T Brignardello-Petersen R Singh JA. A GRADE working group approach for rating the quality of treatment effect estimates from network meta-analysis. Br Med J. (2014) 349:g5630. 10.1136/bmj.g5630 25252733

[13] Brignardello-Petersen R Bonner A Alexander PE Siemieniuk RA Furukawa TA Rochwerg B. Advances in the GRADE approach to rate the certainty in estimates from a network meta-analysis. J Clin Epidemiol. (2018) 93:36–44. 10.1016/j.jclinepi.2017.10.005 29051107

[14] White AR Resch KL Chan JC Norris CD Modi SK Patel JN. Acupuncture for episodic tension-type headache: a multicentre randomized controlled trial. Cephalalgia. (2000) 20(7):632–7. 10.1111/j.1468-2982.2000.00097.x 11128820

[15] Melchart D Streng A Hoppe A Brinkhaus B Witt C Wagenpfeil S. Acupuncture in patients with tension-type headache: randomised controlled trial. Br Med J. (2005) 331(7513):376–82. 10.1136/bmj.38512.405440.8F 16055451 PMC1184247

[16] Endres HG Böwing G Diener HC Lange S Maier C Molsberger A. Acupuncture for tension-type headache: a multicentre, sham-controlled, patient-and observer-blinded, randomised trial. J Headache Pain. (2007) 8(5):306–14. 10.1007/s10194-007-0416-5 17955168 PMC3476149

[17] Zheng H Gao T Zheng QH Lu LY Hou TH Zhang SS. Acupuncture for patients with chronic tension-type headache: a randomized controlled trial. Neurology. (2022) 99(14):e1560–9. 10.1212/WNL.0000000000200670 35732505

[18] Karst M Rollnik JD Fink M Reinhard M Piepenbrock S. Pressure pain threshold and needle acupuncture in chronic tension-type headache–a double-blind placebo-controlled study. Pain. (2000) 88(2):199–203. 10.1016/S0304-3959(00)00315-8 11050375

[19] Karst M Reinhard M Thum P Wiese B Rollnik J Fink M. Needle acupuncture in tension-type headache: a randomized, placebo-controlled study. Cephalalgia. (2001) 21(6):637–42. 10.1046/j.1468-2982.2001.00198.x 11531895

[20] Vickers AJ Cronin AM Maschino AC Lewith G MacPherson H Foster NE. Acupuncture for chronic pain: individual patient data meta-analysis. Arch Intern Med. (2012) 172(19):1444–53. 10.1001/archinternmed.2012.3654 22965186 PMC3658605

[21] Lund I Näslund J Lundeberg T. Minimal acupuncture is not a valid placebo control in randomised controlled trials of acupuncture: a physiologist’s perspective. Chin Med. (2009) 4:1. 10.1186/1749-8546-4-1 19183454 PMC2644695

[22] Lundeberg T Lund I Sing A Näslund J. Is placebo acupuncture what it is intended to be? Evid Based Complement Alternat Med. (2011) 2011:932407. 10.1093/ecam/nep049 19525330 PMC3139519

[23] Yoon DE Lee IS Chae Y. Identifying dose components of manual acupuncture to determine the dose-response relationship of acupuncture treatment: a systematic review. Am J Chin Med. (2022) 50(3):653–71. 10.1142/S0192415X22500264 35300569

[24] Zheng X Yu S Liu L Yang H Wang F Yang H. The dose-related efficacy of acupuncture on endometrial receptivity in infertile women: a systematic review and meta-analysis. Front Public Health. (2022) 10:858587. 10.3389/fpubh.2022.858587 35570887 PMC9095926

[25] Linde K Allais G Brinkhaus B Fei Y Mehring M Shin BC. Acupuncture for the prevention of tension-type headache. Cochrane Database Syst Rev. (2016) 4:CD007587. 10.1002/14651858.CD007587.pub2 27092807 PMC4955729

[26] Lee YS Kim SY Lee H Chae Y Lee MS. ACURATE: a guide for reporting sham controls in trials using acupuncture. J Evid Based Med. (2023) 16(1):82–90. 10.1111/jebm.12524 36959765

