# Supplementary material for: Is the location of the needling point in sham acupuncture for tension-type headache associated with outcomes? A systematic review and network meta-analysis
Source: Front Pain Res (Lausanne). 2026 Aug 6;7:1874475. doi: 10.3389/fpain.2026.1874475 (PMC13492395; doi:10.3389/fpain.2026.1874475)
Supplement: Supplementary file 1 [file Supplementaryfile2.docx]

Supplementary Contents

[Supplement 1. Search strategy used in each database	2](#_Toc216257635)

[Supplement 2. Excluded studies after full-text review	6](#_Toc216257636)

[Supplement 3. Details of acupuncture method	15](#_Toc216257637)

[Supplement 4. Results of testing the inconsistency at the local level through the node splitting method	17](#_Toc216257638)

[Supplement 5. Results of the loop inconsistency test	18](#_Toc216257639)

[Supplement 6. SUCRA plots	19](#_Toc216257640)

[Supplement 7. The certainty of the evidence	21](#_Toc216257641)

# Supplement 1. Search strategy used in each database

**CNKI**

|  | Searches | Results |
| --- | --- | --- |
| #1 | (SU=’紧张型头痛’+’压力性头痛’+’血管性头痛’+’特发性头痛’+’头痛’) AND (SU=’针刺’ +’针灸’) | **1195** |

**Wanfang data**

|  | Searches | Results |
| --- | --- | --- |
| #1 | (题名或关键词:紧张型头痛 OR 压力性头痛 OR 血管性头痛 OR 特发性头痛 OR 头痛) AND (题名或关键词:针刺 OR 针灸) | **7642** |

**Chongqin VIP**

|  | Searches | Results |
| --- | --- | --- |
| #1 | M=(紧张型头痛 OR 压力性头痛 OR 血管性头痛 OR 特发性头痛 OR 头痛) AND M=(针刺 OR 针灸) | **3093** |

**Chinese Biomedical Literature Database (CBM)**

|  | Searches | Results |
| --- | --- | --- |
| #1 | ( "紧张型头痛"[标题] OR "压力性头痛"[标题] OR "血管性头痛"[标题] OR "特发性头痛"[标题] OR "头痛"[标题])AND( "针刺"[标题] OR "针灸"[标题]) | **2065** |

**MEDLINE via PubMed**

|  | Searches | Results |
| --- | --- | --- |
| #1 | "Tension-Type Headache"[TIAB] OR "TTH"[TIAB] OR "idiopathic headache"[TIAB] OR "stress headache"[TIAB] OR "psychogenic headache"[TIAB] OR "tension vascular headache"[TIAB] OR "tension headache"[TIAB] OR "headache"[TIAB] OR "headache disorder"[TIAB] OR "head pain"[TIAB] OR "cephalalgia"[TIAB] OR "cephalodynia"[TIAB] OR "cranial pain"[TIAB] OR "cerebral pain"[TIAB] OR "Tension-Type Headache"[MH] | 100,406 |
| #2 | Acupuncture[MH] OR “Acupuncture Therapy”[MH] OR “Acupuncture Points”[MH] OR acupunct*[TIAB] OR acupoint*[TIAB] OR “Dry Needling”[MH] OR “dry needling”[TIAB] OR "filiform needle"[TIAB] | 44,472 |
| #3 | “Randomized Controlled Trial”[PT] OR “Controlled Clinical Trial”[PT] OR randomized[TIAB] OR placebo[TIAB] OR “Clinical Trials as Topic”[Mesh: noexp] OR randomly[TIAB] OR trial[TI] | 1,816,334 |
| #4 | animals[MH] NOT humans[MH] | 5,379,443 |
| #5 | (#1 AND #2 AND #3) NOT #4 | **392** |

**Embase via Elsevier.com**

|  | Searches | Results |
| --- | --- | --- |
| #1 | 'tension-type headache':ab,ti OR tth:ab,ti OR 'idiopathic headache':ab,ti OR 'stress headache':ab,ti OR 'psychogenic headache':ab,ti OR 'tension-vascular headache':ab,ti OR 'tension headache':ab,ti OR headache:ab,ti OR 'headache disorder':ab,ti OR 'head pain':ab,ti OR cephalalgia:ab,ti OR cephalodynia:ab,ti OR 'cranial pain':ab,ti OR 'cerebral pain':ab,ti OR 'tension headache'/exp | 166,657 |
| #2 | acupuncture/exp OR acupuncture*:ab,ti OR ‘acupuncture point’/exp OR ‘body meridian’/exp OR ‘body meridian’:ab,ti OR acupoint*:ab,ti OR 'dry needling'/exp OR 'dry needling':ab,ti OR ‘filiform needle’:ab,ti | 75,128 |
| #3 | 'crossover procedure':de OR 'double-blind procedure':de OR 'randomized controlled trial':de OR 'single-blind procedure':de OR (random* OR factorial* OR crossover* OR cross NEXT/1 over* OR placebo* OR doubl* NEAR/1 blind* OR singl* NEAR/1 blind* OR assign* OR allocat* OR volunteer*):de,ab,ti | 3,882,896 |
| #4 | #1 AND #2 AND #3 | **825** |

**CENTRAL**

|  | Searches | Results |
| --- | --- | --- |
| #1 | MeSH descriptor: [Tension-Type Headache] explode all trees | 425 |
| #2 | ((tension-type headache) OR TTH OR (idiopathic headache) OR (stress headache) OR (psychogenic headache) OR (tension-vascular headache) OR (tension headache) OR headache OR (headache disorder) OR (head pain) OR cephalalgia OR cephalodynia OR (cranial pain) OR (cerebral pain)):ti,ab,kw | 45,943 |
| #3 | #1 OR #2 | 46,119 |
| #4 | MeSH descriptor: [Acupuncture] explode all trees | 216 |
| #5 | MeSH descriptor: [Acupuncture Therapy] explode all trees | 7,267 |
| #6 | MeSH descriptor: [Acupuncture Points] explode all trees | 2,886 |
| #7 | MeSH descriptor: [Dry Needling] explode all trees | 225 |
| #8 | (acupunct* OR acupoint* OR “dry needling” OR "filiform needle"):ti,ab,kw | 25,677 |
| #9 | #4 OR #5 OR #6 OR #7 OR #8 | 26,063 |
| #10 | (#3 AND #9) in Trials | **1028** |

**AMED via EBSCO**

|  | Searches | Results |
| --- | --- | --- |
| #1 | SU Tension-Type Headache OR TX tension-type headache OR TX TTH OR TX idiopathic headache OR TX stress headache OR TX psychogenic headache OR TX tension-vascular headache OR TX tension headache OR TX headache OR TX headache disorder OR TX head pain OR TX cephalalgia OR TX cephalodynia OR TX cranial pain OR TX cerebral pain | 2,230 |
| #2 | SU Acupuncture OR SU Acupuncture Therapy OR SU Acupuncture Points ORTX acupunct* OR TX acupoint* OR SU Dry Needling OR TX dry needling OR TX filiform needle | 10,711 |
| #3 | #1 AND #2 | **310** |

# Supplement 2. Excluded studies after full-text review

**- Not randomized controlled trials: 13**

[1] 樊雪鸣,申伟,魏竞竞,付国静,梁晓,宫晓,陈文洁,朴京泽,闫羽,郭春莉,滕晶,张慧,张允岭.针刺治疗紧张型头痛临床研究的概括性评价[J].中医杂志,2022,63(6):572-580
[2] 邓赣,罗晓舟,王梓楠,等. 针灸治疗紧张性头痛的临床疗效及安全性分析[J]. 中国老年学杂志,2018,38(12):2936-2940.
[3] 赵海音,高明清. 针刺治疗紧张型头痛临床观察[J]. 上海针灸杂志,2009,28(12):711-712.
[4] 李漾,罗纯.针刺治疗紧张性头痛疗效的系统评价[J].中国循证医学杂志,2005,5(2):117-124
[5] 袁保丰.针刺为主治疗紧张性头痛109例[J].河北中医,2004,(10):770.
[6] 钟纪萍,黄曼华.针刺治疗紧张性头痛50例[J].宜春医专学报,2001,(01):86-87.
[7] 马朱红,王荣春,张晓燕. 针灸治疗紧张性头痛64例临床观察[J]. 浙江中医学院学报,1997,
[8] Cao, W., Wang, L., Hou, T. H., Shi, Y. Z., Zheng, Q. H., Zheng, H., Zou, Z. H., Qin, D., Yang, Q., Chen, S. J., Wang, H. Y., Xiao, X. J., & Li, Y. (2024). Disease-Related Factors Associated with Acupuncture Response in Patients with Chronic Tension-Type Headache: A Secondary Analysis of A Randomized Controlled Trial. Chinese journal of integrative medicine, 30(8), 684–691.
[9] Lu, L., Zheng, H., Zheng, Q., Hao, X., Zhou, S., Zhang, S., Wei, T., Gao, T., Duan, D., Zhao, L., Li, N., & Li, Y. (2017). The long-term effect of acupuncture for patients with chronic tension-type headache: study protocol for a randomized controlled trial. Trials, 18(1), 453.
[10] Schiller J., Buttner A., Niederer D., Bokel A., Korallus C., Sturm C., Vogt L., Gutenbrunner C., Karst M., Fink M., Egen C. (2024). Effects on temporomandibular disorder in the treatment of tension-type headache with acupuncture and therapeutic exercises. A secondary analysis from a randomized controlled trial. Clinical rehabilitation, 38(5), 623-635.
[11] Callison M. (2008). The treatment of sports-related muscle tension headaches with acupuncture and moxibustion. Journal of Chinese Medicine, 87, 34–38.
[12] Tukmachi E, & Tukmachi F. (2005). Tension headache: A clinical study of its treatment by acupuncture. Journal of Chinese Medicine, 78, 10–16.
[13] Melchart D, Linde K, Streng A, Reitmayr S, Hoppe A, Brinkhaus B, Becker-Witt C, Wagenpfeil S, Pfaffenrath V, Hammes M, Willich SN, & Weidenhammer W. (2003). Acupuncture randomized trials (ART) in patients with migraine or tension-type headache - design and protocols. Forsch Komplementarmed Klass Naturheilkd, 10(4), 179–184.

**- Not about TTH patients: 3**

[1] Jensen, L. B., Melsen, B., & Jensen, S. B. (1979). Effect of acupuncture on headache measured by reduction in number of attacks and use of drugs. Scandinavian journal of dental research, 87(5), 373–380.
[2] He Q.-Y., Liang J., Zhang Y., Zhang J. (2009). Thirty-two cases of vascular headache treated by acupuncture combined with Chinese herbal decoction. Journal of Traditional Chinese Medicine, 29(4), 253-257.
[3] Tang W. (2002). Clinical observation on scalp acupuncture treatment in 50 cases of headache. Journal of Traditional Chinese Medicine, 22(3), 190–192.

**- Not only about manual acupuncture: 19**

[1] 孟冬祥,樊碧发,周书元,等. 传统针刺与安慰针刺治疗紧张性头痛的初步比较研究[J]. 疼痛学杂志,1996, (1):6-7.
[2] Guo, N. N., & Shen, H. Q. (2020). Zhen ci yan jiu = Acupuncture research, 45(2), 148–151.
[3] Xue, C. C., Dong, L., Polus, B., English, R. A., Zheng, Z., Da Costa, C., Li, C. G., & Story, D. F. (2004). Electroacupuncture for tension-type headache on distal acupoints only: a randomized, controlled, crossover trial. Headache, 44(4), 333–341.
[4] Wang, K., Svensson, P., & Arendt-Nielsen, L. (2007). Effect of acupuncture-like electrical stimulation on chronic tension-type headache: a randomized, double-blinded, placebo-controlled trial. The Clinical journal of pain, 23(4), 316–322.
[5] Chassot, M., Dussan-Sarria, J. A., Sehn, F. C., Deitos, A., de Souza, A., Vercelino, R., Torres, I. L., Fregni, F., & Caumo, W. (2015). Electroacupuncture analgesia is associated with increased serum brain-derived neurotrophic factor in chronic tension-type headache: a randomized, sham controlled, crossover trial. BMC complementary and alternative medicine, 15, 144.
[6] Airaksinen, O., & Põntinen, P. J. (1992). Effects of the electrical stimulation of myofascial trigger points with tension headache. Acupuncture & electro-therapeutics research, 17(4), 285–290.
[7] A, R., Bao, Q., Sai, Y. C., Te, M., Hu, R., Sa, R., Mu, R., & Bo, A. (2021). Clinical observation of warming acupuncture and moxibustion at the temples combined with Deanxit in the treatment of tension headache with anxiety and depression: a retrospective study. Annals of palliative medicine, 10(5), 5541–5548.
[8] Gildir, S., Tüzün, E. H., Eroğlu, G., & Eker, L. (2019). A randomized trial of trigger point dry needling versus sham needling for chronic tension-type headache. Medicine, 98(8), e14520.
[9] Vincent C. A. (1990). The treatment of tension headache by acupuncture: a controlled single case design with time series analysis. Journal of psychosomatic research, 34(5), 553–561.
[10] Son J.-Y., Goo K., Kim N.-Y., Yang S.-G., Lee D.H., Im Y.-R., Kwon O.B., Cho H.-W., Kim S.D., Kim D., Ha I.-H. (2024). Effectiveness and Safety of Pharmacopuncture on Inpatients with Tension Headache Caused by Traffic Accidents: A Pragmatic Randomized Controlled Trial. Journal of Clinical Medicine, 13(15).
[11] Immune-Pineal Axis Function in Chronic Tension-Type Headache: Noradrenergic Modulation and the Effect of Electroacupuncture (2013), Identifier: NCT01954277.
[12] To Explore the Cerebral Function Efficacy of Acupuncture and Fu's Subcutaneous Needling (FSN) in the Treatment of Chronic Tension-type Headache by Means of fMRI: a Single-blinded, Randomized Controlled Trials. (2017), Identifier: NCT03171337.
[13] Pei F., Hu W.-J., Zhao Y.-L. (2023). Efficacy of Jiao's scalp acupuncture in combination with Xiangshao granules on chronic tension-type headache with concomitant nonalcoholic fatty liver disease in climacteric women. Menopause, 30(11), 1132-1138.
[14] The Classification of Myofascial Headache: a Cross-sectional Study. the Added Value of Dry Needling in the Treatment of Myofascial Headache: a Randomized Controlled Trial. (2023), Identifier: NCT06100588.
[15] Comparing the Effectiveness of Dry Needling and Ischemic Compression Technique on Muscle Thickness and Health Related Quality of Life in Trigger Point Tension Type Headache (2022), Identifier: NCT05496010.
[16] Koran S., Sang J.E., Burcu P., Duz O.A., Sarihan A. (2021). Search for the clinical effectiveness of Korean Tae-Geuk acupuncture therapy in chronic tension-type headache. Revista Internacional de Acupuntura, 15(4).
[17] Karakurum B., Karaalin O., Coskun O., Dora B., Ucler S., Inan L.E. (2001). The 'dry-needle technique': Intramuscular stimulation in tension-type headache. Cephalalgia, 21(8), 813-817.
[18] Ebneshahidi NS, Heshmatipour M, Moghaddami A, & Eghtesadi-Araghi P. (2005). The effects of laser acupuncture on chronic tension headache - a randomised controlled trial. Acupuncture in Medicine, 23(1), 13–18.
[19] Lavies NG. (1998). Laser acupuncture for migraine and muscle tension headache: a double-blind controlled trial. Acupuncture in Medicine, 16(2), 73–76.

**- Not sham acupuncture or waiting list-controlled trials: 36**

[1] 陈易,孟建霞,岳艳芳,等. 针刺治疗紧张性头痛临床观察[J]. 光明中医,2022,37(9):1616-1618.
[2] 周卫星. 针灸对紧张性头痛的临床治疗效果观察[J]. 大医生,2020,5(15):36-38.
[3] 邵晨旭,张立德.针灸对头痛治疗的临床研究[J].中医临床研究,2019,11(15):84-86.
[4] 魏韬,张树森,鲁凌云,郑倩华,周思远,李瑛.针刺治疗慢性紧张型头痛临床疗效与期待值的相关性研究[J].世界中医药,2018,13(7):1588-1591
[5] 尤阳. 针刺治疗慢性紧张性头痛的临床观察[C]//中国中医药研究促进会针灸康复分会第二届学术年会暨山东针灸学会第九届学术年会论文集. 2017:341-345.
[6] 陈新昌,冯均信. 针刺治疗发作性紧张型头痛临床疗效观察[J]. 陕西中医药大学学报,2017,40(6):86-88.
[7] 吴昕. 针刺调神方治疗紧张性头痛的临床观察[D]. 广东:广州中医药大学,2016.
[8] 李文龙.针刺治疗紧张型头痛的临床观察[J].中国民间疗法,2016,24(5):16-16
[9] 段珊. 针刺治疗紧张型头痛48例临床疗效观察[J]. 中国民族民间医药,2014,23(1):54-54.
[10] 朱晓蕾. 针刺治疗紧张型头痛的临床研究[D]. 湖北:湖北中医药大学,2013.
[11] 付梅.针刺治疗紧张型头痛的临床观察[J].中国中医基础医学杂志,2013,19(07):810-811.
[12] 黄淑珍. 针刺治疗紧张型头痛的临床观察[D]. 江苏:南京中医药大学,2012.
[13] 雷行华,何继原. 针灸治疗慢性紧张型头痛疗效分析[J]. 四川中医,2011,29(9):114-115.
[14] 张路,张琰,刘慧林,等. 针刺治疗慢性紧张型头痛随机对照研究[C]//2009年全国针灸临床学术研讨会暨北京地区针灸名家学术推广会论文集. 2009:32-36.
[15] 张路. 针刺治疗慢性紧张型头痛的临床观察[D]. 北京:首都医科大学,2009.
[16] 彭建民. 针刺治疗紧张性头痛的临床研究[J]. 中医药学报,2009,37(2):47-48.
[17] 陈奇才.针刺治疗紧张性头痛疗效观察[J].实用中医药杂志,2008,(05):312-313.
[18] 江勇,王敏华,王敏,等. 针刺推拿治疗紧张性头痛临床研究[J]. 针灸临床杂志,2003,19(4):7-8.
[19] 岳进. 针刺治疗偏头痛、紧张性头痛的临床观察[J]. 上海针灸杂志,1992, (3):15.
[20] 李国辉,刘梅.针刺治疗紧张性头痛40例[J].中国民间疗法,2012,20(05):9-10.
[21] Schiller, J., Niederer, D., Kellner, T., Eckhardt, I., Egen, C., Zheng, W., Korallus, C., Achenbach, J., Ranker, A., Sturm, C., Vogt, L., Gutenbrunner, C., Fink, M. G., & Karst, M. (2023). Effects of acupuncture and medical training therapy on depression, anxiety, and quality of life in patients with frequent tension-type headache: A randomized controlled study. Cephalalgia : an international journal of headache, 43(1), 3331024221132800.
[22] Carlsson, J., Fahlcrantz, A., & Augustinsson, L. E. (1990). Muscle tenderness in tension headache treated with acupuncture or physiotherapy. Cephalalgia : an international journal of headache, 10(3), 131–141.
[23] Söderberg, E., Carlsson, J., & Stener-Victorin, E. (2006). Chronic tension-type headache treated with acupuncture, physical training and relaxation training. Between-group differences. Cephalalgia : an international journal of headache, 26(11), 1320–1329.
[24] Schiller, J., Karst, M., Kellner, T., Zheng, W., Niederer, D., Vogt, L., Eckhardt, I., Beissner, F., Korallus, C., Sturm, C., Egen, C., Gutenbrunner, C., & Fink, M. G. (2021). Combination of acupuncture and medical training therapy on tension type headache: Results of a randomised controlled pilot study. Cephalalgia : an international journal of headache, 41(8), 879–893.
[25] Carlsson, J., Augustinsson, L. E., Blomstrand, C., & Sullivan, M. (1990). Health status in patients with tension headache treated with acupuncture or physiotherapy. Headache, 30(9), 593–599.
[26] Carlsson, J., & Rosenhall, U. (1990). Oculomotor disturbances in patients with tension headache treated with acupuncture or physiotherapy. Cephalalgia : an international journal of headache, 10(3), 123–129.
[27] Monti-Ballano, S., Márquez-Gonzalvo, S., Lucha-López, M. O., Ferrández-Laliena, L., Vicente-Pina, L., Sánchez-Rodríguez, R., Tricás-Vidal, H. J., & Tricás-Moreno, J. M. (2024). Effects of Dry Needling on Active Myofascial Trigger Points and Pain Intensity in Tension-Type Headache: A Randomized Controlled Study. Journal of personalized medicine, 14(4), 332.
[28] Söderberg, E. I., Carlsson, J. Y., Stener-Victorin, E., & Dahlöf, C. (2011). Subjective well-being in patients with chronic tension-type headache: effect of acupuncture, physical training, and relaxation training. The Clinical journal of pain, 27(5), 448–456.
[29] Dowson, D. I., Lewith, G. T., & Machin, D. (1985). The effects of acupuncture versus placebo in the treatment of headache. Pain, 21(1), 35–42.
[30] Chen, R. H., Chen, R. L., & Chen, R. F. (2013). Zhongguo zhen jiu = Chinese acupuncture & moxibustion, 33(3), 219–222.
[31] Schiller, J., Kellner, T., Briest, J., Hoepner, K., Woyciechowski, A., Ostermann, A., Korallus, C., Sturm, C., Weiberlenn, T., Jiang, L., Egen, C., Beissner, F., Stiesch, M., Karst, M., Gutenbrunner, C., & Fink, M. G. (2019). The best from East and West? Acupuncture and medical training therapy as monotherapies or in combination for adult patients with episodic and chronic tension-type headache: study protocol for a randomized controlled trial. Trials, 20(1), 623.
[32] Georgoudis, G., Felah, B., Nikolaidis, P., & Damigos, D. (2018). The effect of myofascial release and microwave diathermy combined with acupuncture versus acupuncture therapy in tension-type headache patients: A pragmatic randomized controlled trial. Physiotherapy research international : the journal for researchers and clinicians in physical therapy, 23(2), e1700.
[33] Olivier S., Antonioni A., Mezzetta B., Capone J.G., Pugliatti M., Granieri E. (2022). A trial to prove the efficacy of acupuncture as a therapeutic support in pharmacological prophylaxis for migraine and tension-type headache. Pilot study. Confinia Cephalalgica et Neurologica, 32(1).
[34] Georgoudis G., Felah B., Nikolaidis P.T., Papandreou M., Mitsiokappa E., Mavrogenis A.F., Rosemann T., Knechtle B. (2018). The effect of physiotherapy and acupuncture on psychocognitive, somatic, quality of life, and disability characteristics in TTH patients. Journal of Pain Research, 11, 2527-2535.
[35] Silva J.B.G., Nakamura M.U., Cordeiro J.A., Kulay Jr. L. (2013). Acupuncture for tension-type headache in pregnancy: A prospective, randomized, controlled study, Society for Acupuncture Research 2013 International Conference: Impact of Acupuncture Research on 21st Century Healthcare. Journal of Alternative and Complementary Medicine.
[36] Kamali F, Mohamadi M, Fakheri L, & Mohammadnejad F. (2019). Dry needling versus friction massage to treat tension type headache: A randomized clinical trial. Journal of Bodywork and Movement Therapies, 23(1), 89–93.

- **Not adequately reporting the outcome of interest: 6**

[1] 周健.传统中国针刺治疗紧张性头痛:对照研究[J].国外医学(中医中药分册),1994,(01):55.
[2] Hong K ,Parkb Y ,Jo J , et al.假针灸治疗慢性紧张性头痛疗效的随机对照实验[C]//世界针灸学会联合会,世界卫生组织,中国中医科学院,北京市中医管理局.世界针灸学会联合会成立20周年暨世界针灸学术大会论文摘要汇编.Department of Acupuncture and Moxibustion,College of Oriental Medicine,Daejeon University,Daejeon,Korea;Department of Acupuncture and Moxibustion,College of Oriental Medicine,Daejeon University,Daejeon,Korea;Department of Acupuncture and Moxibustion,College of Oriental Medicine,Daejeon University,Daejeon,Korea;Department of Neuropsychology,College of Oriental Medicine,Daejeon University,Daejeon,Korea;Department of Applicative Statistics,College of Economics,Daejeon University,Daejeon,Korea;Department of Neurology,College of Medicine,Catholic Medicine University,Daejeon,Korea;Korea Institute of Oriental Medicine,Daejeon,Korea;,2007:497-498.
[3] 梁瑞华,张素平,何锐.辩证取穴针刺治疗对慢性紧张型头痛中枢调控的影响[C]//中华医学会（Chinese Medical Association）,中华医学会神经病学分会（Chinese Society of Neurology）.中华医学会第十八次全国神经病学学术会议论文汇编（下）.广州市红十字会医院神经内科;,2015:439.
[4] Tavola, T., Gala, C., Conte, G., & Invernizzi, G. (1992). Traditional Chinese acupuncture in tension-type headache: a controlled study. Pain, 48(3), 325–329.
[5] Hansen, P. E., & Hansen, J. H. (1985). Acupuncture treatment of chronic tension headache--a controlled cross-over trial. Cephalalgia : an international journal of headache, 5(3), 137–142.
[6] White A.R., Eddleston C., Hardie R., Resch K.L., Ernst E. (1996). A pilot study of acupuncture for tension headache, using a novel placebo. Acupuncture in Medicine, 14(1), 11-15.

**- Duplicate data: 2**

[1] Melchart D.,Streng A.,Hoppe A.,郭俊.针刺治疗紧张型头痛患者的随机、对照试验[J].世界核心医学期刊文摘（神经病学分册）,2005,0(12):1-1
[2] Melchart D., Streng A., Hoppe A., Linde K., Brinkhaus B., Becker-Witt C., Willich S.N., Hammes M., Irnich D., Hummelsberger J. (2005). The acupuncture randomised trial (ART) for tension-type headache - Details of the treatment. Acupuncture in Medicine, 23(4), 157-165.

# Supplement 3. Details of acupuncture method

| Study ID | Acupuncture protocol | Treatment points | Depth of insertion | Needle stimulation | Needle retention time | Needle type (number of needle) | Number of treatment session | Frequency | Qualification or experiences on acupuncture | Other interventions in all groups | Study setting | Funding source |
| --- | --- | --- | --- | --- | --- | --- | --- | --- | --- | --- | --- | --- |
| AR White, 2000 | Semi-standardized | bilateral GB20, LI4 | not predefined (NR) | De qi, manually stimulated | 15s | 30 x 0.30 mm (1) | 6 | once a week for 6 weeks | the practitioner in the British Medical Acupuncture Society | take one named analgesic, if required | Department of Complementary Medicine at the University of Exeter | the United Kingdom's NHS Executive South-west R & D |
| Dieter Melchart, 2005 | Semi-standardized | Basic points and additional points (from a prescribed list) chosen individually | NR | De qi, manually stimulated | 30min | free in choice of length and diameter of needle (25) | 12 | two sessions in each of the first four weeks, followed by one session a week in the remaining four weeks | Physicians trained (at least 140 hours, median 500 hours) and experienced (median 10 years) in acupuncture | allowed to treat acute headaches as needed | 28 outpatient centers in Germany | German Social Health Insurance Companies |
| Heinz G. Endres, 2007 | Semi-standardized | decided by acupuncturist | 2–30 mm | De qi, manually stimulated | 30min | 0.25–0.30 mm thick, 25–40 mm long (10–25) | 10 | twice a week | All physicians had at least 140 h of acupuncture training: 43% had received basic training (mean 208 h) and 57% had advanced training (mean 398 h) | allowed to take only one of their pre-baseline oral headache analgesics | 122 family physician practices in 11 federal states in Germany | German public health insurance companies: AOK, BKK, IKK, Bundesknappschaft, Bundesverband der Landwirtschaftlichen Krankenkassen and Seekasse |
| Hui Zheng, 2022 | Standardized | bilateral GB20, GV20, EX-HN5, LI4, LR3 | 12.5 to 20 mm | De qi, manually stimulated | 30min | NR | 20 | 3 sessions per week in the first 4 weeks and 2 sessions per week in the last 4 weeks | Acupuncturists had at least 5 years of training and had experience in participating in clinical trials | Headache education in each clinic visit | outpatient settings of the Teaching Hospital of Chengdu Uni- versity of Traditional Chinese Medicin | the Department of Science and Technology of Sichuan Province; the National Natural Science Foundation of China |
| Matthias Karst, 2000 | Individualized | GB20, LI4, LR3, GB8, GB14, GB21, GB41, UB2, UB10, UB60, LU7, TW5, ST8, ST36, ST44, DU20, Extra 1 | NR | NR | 30min | 0.3 x 0.3 mm and 0.2 x 0.15 mm (A maximum of 15 needles) | 10 | 5 weeks twice a week | NR | Concomitant medication was allowed (including analgesics and rescue medications) | NR | NR |
| M Karst, 2001 | Individualized | GB20, LI4, LR3, GB8, GB14, GB21, GB41, UB2, UB10, UB60, LU7, TW5, ST8, ST36, ST44, DU20, Extra 1 | NR | NR | 30min | 0.3 x 0.3 mm and 0.2 x 0.15 mm (A maximum of 15 needles) | 10 | 5 weeks twice a week | NR | Concomitant medication was allowed (including analgesics and rescue medications) | NR | NR |

# Supplement 4. Results of testing the inconsistency at the local level through the node splitting method

| Outcome | Side | Direct | | Indirect | | Difference | | P value |
| --- | --- | --- | --- | --- | --- | --- | --- | --- |
|  |  | Coefficient | SE | Coefficient | SE | Coefficient | SE |  |
| Pain intensity | AT WL | -0.580949 | 0.1580939 | -1.32968 | 0.6465689 | 0.7487316 | 0.6528628 | 0.251 |
|  | SATS WL | -0.670326 | 0.184579 | 0.0784294 | 0.6251632 | -0.7487553 | 0.6528663 | 0.251 |
|  | AT SATV | -0.078181 | 0.1108862 | 1.144816 | 391.1694 | -1.222997 | 391.1694 | 0.998 |
|  | AT SATS | 0.000781 | 0.1395773 | -1.217141 | 354.7645 | 1.217922 | 354.7645 | 0.997 |
| Headache days | AT WL | -0.612132 | 0.1585901 | -0.760408 | 0.3621004 | 0.1482763 | 0.3744396 | 0.692 |
|  | SATS WL | -0.547209 | 0.1845501 | -0.398927 | 0.3230996 | -0.1482819 | 0.3744401 | 0.692 |
|  | AT SATV | 0.1898316 | 0.118354 | 1.4468 | 425.8636 | -1.256968 | 425.8636 | 0.998 |
|  | AT SATS | 0.1199758 | 0.0818581 | -1.143547 | 471.494 | 1.263523 | 471.494 | 0.998 |
| Response rate | AT WL | 2.446926 | 0.5734198 | 2.280094 | 0.6874394 | 0.1668323 | 0.4231321 | 0.693 |
|  | SATS WL | 2.166761 | 0.591251 | 2.333575 | 0.6405393 | -0.1668131 | 0.4231336 | 0.693 |
|  | AT SATV | -0.44827 | 0.1600813 | -5.38656 | 729.0751 | 4.938289 | 729.0751 | 0.995 |
|  | AT SATS | -0.208608 | 0.0738738 | 4.614638 | 121.8924 | -4.823247 | 121.8924 | 0.968 |

Abbreviations: AT, acupuncture therapy; SATV, sham acupuncture needling at the same acupuncture points as those in the acupuncture group, referred to as sham acupuncture therapy (verum); SATS, sham acupuncture needling at points different from those in the acupuncture group, referred to as sham acupuncture therapy (sham); WL, waiting list.

# Supplement 5. Results of the loop inconsistency test

| Outcome | Loop | IF | P value | CI_95 |
| --- | --- | --- | --- | --- |
| Pain intensity | AT-SATS-WL | 0.374 | 0.317 | (0.00,1.11) |
| Headache days | AT-SATS-WL | 0.074 | 0.777 | (0.00,0.59) |
| Response rate | AT-SATS-WL | 0.083 | 0.92 | (0.00,1.71) |

Abbreviations: AT, acupuncture therapy; SATS, sham acupuncture needling at points different from those in the acupuncture group, referred to as sham acupuncture therapy (sham); WL, waiting list.

# Supplement 6. SUCRA plots

(A) Pain intensity

(B) Headache days

(C) Response rate

Abbreviations: AT, acupuncture therapy; SATV, sham acupuncture needling at the same acupuncture points as those in the acupuncture group, referred to as sham acupuncture therapy (verum); SATS, sham acupuncture needling at points different from those in the acupuncture group, referred to as sham acupuncture therapy (sham); WL, waiting list.

# Supplement 7. The certainty of the evidence

| Comparison | | Direct evidence | Indirect evidence | Network meta-analysis |
| --- | --- | --- | --- | --- |
| **Pain intensity** | | | | |
| AT | SATV | Moderate Risk of bias (-1) | - | Low Risk of bias (-1) Imprecision (-1) |
| AT | SATS | Moderate Risk of bias (-1) | Moderate Risk of bias (-1) | Low Risk of bias (-1) Imprecision (-1) |
| AT | WL | Moderate Risk of bias (-1) | Moderate Risk of bias (-1) | Moderate Risk of bias (-1) |
| SATS | WL | Moderate Risk of bias (-1) | Moderate Risk of bias (-1) | Moderate Risk of bias (-1) |
| SATS | SATV | - | Moderate Risk of bias (-1) | Low Risk of bias (-1) Imprecision (-1) |
| SATV | WL | - | Moderate Risk of bias (-1) | Moderate Risk of bias (-1) |
| **Headache days** | | | | |
| AT | SATV | Moderate Risk of bias (-1) | - | Low Risk of bias (-1) Imprecision (-1) |
| AT | SATS | Moderate Risk of bias (-1) | Moderate Risk of bias (-1) | Low Risk of bias (-1) Imprecision (-1) |
| AT | WL | Moderate Risk of bias (-1) | Moderate Risk of bias (-1) | Moderate Risk of bias (-1) |
| SATS | WL | Moderate Risk of bias (-1) | Moderate Risk of bias (-1) | Moderate Risk of bias (-1) |
| SATS | SATV | - | Moderate Risk of bias (-1) | Low Risk of bias (-1) Imprecision (-1) |
| SATV | WL | - | Moderate Risk of bias (-1) | Moderate Risk of bias (-1) |
| **Response rate** | | | | |
| AT | SATV | High | - | High |
| AT | SATS | Moderate Risk of bias (-1) | Moderate Risk of bias (-1) | Moderate Risk of bias (-1) |
| AT | WL | Moderate Risk of bias (-1) | Moderate Risk of bias (-1) | Moderate Risk of bias (-1) |
| SATS | WL | Moderate Risk of bias (-1) | Moderate Risk of bias (-1) | Moderate Risk of bias (-1) |
| SATS | SATV | - | Moderate Risk of bias (-1) | Low Risk of bias (-1) Imprecision (-1) |
| SATV | WL | - | Moderate Risk of bias (-1) | Moderate Risk of bias (-1) |

Abbreviations: AT, acupuncture therapy; SATV, sham acupuncture needling at the same acupuncture points as those in the acupuncture group, referred to as sham acupuncture therapy (verum); SATS, sham acupuncture needling at points different from those in the acupuncture group, referred to as sham acupuncture therapy (sham); WL, waiting list.
